# Diagnostik und Therapie der Fokal-Segmentalen Glomerulosklerose – 2023

**DOI:** 10.1007/s00508-023-02260-x

**Published:** 2023-09-20

**Authors:** Philipp Gauckler, Emanuel Zitt, Heinz Regele, Kathrin Eller, Marcus D. Säemann, Karl Lhotta, Irmgard Neumann, Michael Rudnicki, Balazs Odler, Andreas Kronbichler, Johannes Zschocke, Martin Windpessl

**Affiliations:** 1 Department Innere Medizin IV (Nephrologie und Hypertensiologie), Medizinische Universität Innsbruck, Innsbruck, Österreich; 2 Abteilung für Innere Medizin III (Nephrologie, Dialyse und Hypertensiologie), Akademisches Lehrkrankenhaus Feldkirch, Feldkirch, Österreich; 3 Klinisches Institut für Pathologie, Medizinische Universität Wien, Wien, Österreich; 4 Klinische Abteilung für Nephrologie, Abteilung für Innere Medizin III (Nephrologie, Dialyse und Hypertensiologie), Medizinische Universität Graz, Graz, Österreich; 5 6.Medizinische Abteilung mit Nephrologie & Dialyse, Klinik Ottakring, Wien, Österreich; 6 Medizinische Fakultät, SFU, Wien, Österreich; 7 Vasculitis.at, Wien, Österreich; 8 Immunologiezentrum Zürich (IZZ), Zürich, Schweiz; 9 Department Innere Medizin IV (Nephrologie und Hypertensiologie), Medizinische Universität Innsbruck, Innsbruck, Österreich; 10 Klinische Abteilung für Nephrologie, Abteilung für Innere Medizin III (Nephrologie, Dialyse und Hypertensiologie), Medizinische Universität Graz, Graz, Österreich; 11 Department Innere Medizin 4 (Nephrologie und Hypertensiologie), Medizinische Universität Innsbruck, Innsbruck, Österreich; 12 Institut für Humangenetik, Medizinische Universität Innsbruck, Innsbruck, Österreich; 13 Abteilung für Innere Medizin IV, Klinikum Wels-Grieskirchen, Wels, Österreich

**Keywords:** Fokal-segmentale Glomerulosklerose, Proteinurie, Podozytopathie, Glukokortikoide, Rituximab, Supportivtherapie, Focal-segmental glomerulosclerosis, Proteinuria, Podocytopathy, Glucocorticoids, Rituximab, Supportive therapy

## Abstract

Der histopathologische Begriff fokal-segmentale Glomerulosklerose umfasst verschiedene Krankheitsprozesse mit dem gemeinsamen Kennzeichen einer großen Proteinurie und dem namensgebenden glomerulären Schädigungsmuster in der Lichtmikroskopie. Eine Einteilung in *primäre, sekundäre* und *genetische* Formen anhand der zugrundeliegenden Pathogenese ist daher von großer Relevanz. Die exakte Pathogenese der primären fokal-segmentalen Glomerulosklerose ist ungeklärt, allerdings wird – analog zur Minimal-change Glomerulopathie – eine autoimmun-vermittelte Schädigung der Podozyten angenommen. Angesichts des ähnlichen Pathomechanismus findet zunehmend die vereinende Bezeichnung „Podozytopathie“ Verwendung. Supportive Therapiemaßnahmen zum Erhalt der Nierenfunktion sind bei allen Formen angezeigt. Demgegenüber sollten immunsuppressive Therapien nur bei der primären fokal-segmentalen Glomerulosklerose zum Einsatz kommen. Komplizierte Verläufe umfassen steroid-abhängige, steroid-resistente und häufig relapsierende Formen und erfordern den Einsatz alternativer Therapiestrategien. Die Österreichische Gesellschaft für Nephrologie (ÖGN) stellt hier einen gemeinsamen Konsens darüber vor, wie erwachsene PatientInnen mit fokal-segmentaler Glomerulosklerose am besten diagnostiziert und behandelt werden können.

## Abstract (en)

The histopathological term focal-segmental glomerulosclerosis comprises different pathogenic processes with the unifying features of a high proteinuria and the name-giving glomerular lesion pattern seen on light microscopy. A differentiation according to the underlying cause into primary, secondary and genetic forms is therefore of utmost importance. The pathogenesis of primary focal-segmental glomerulosclerosis remains unknown but, like minimal-change disease, an autoimmune-mediated process leading to podocyte damage is assumed. Consequently, the unifying term “podocytopathy” is increasingly being used for both entities. Supportive treatment measures to preserve kidney function are important in all subtypes. In contrast, immunosuppressive treatment is only indicated in primary focal-segmental glomerulosclerosis. Steroid-dependence, steroid-resistance and frequently relapsing disease often complicate disease management and necessitate alternative treatment strategies. Here, the Austrian Society of Nephrology (ÖGN) provides consensus recommendations on how to best diagnose and manage patients with focal-segmental glomerulosclerosis.

## Einleitung und Epidemiologie

Der histopathologische Begriff fokal-segmentale Glomerulosklerose (FSGS) umfasst verschiedene Nephropathien mit dem gemeinsamen Kennzeichen einer großen Proteinurie (meist im nephrotischen Bereich) und dem namensgebenden glomerulären Schädigungsmuster in der Lichtmikroskopie. Dieses ist jedoch unspezifisch und kann durch diverse Prozesse ausgelöst werden. Eine Einteilung in *primäre, sekundäre* und *genetische* Formen anhand der zugrundeliegenden Pathogenese ist daher auch prognostisch und therapeutisch von großer Relevanz. Bei der primären FSGS wird eine autoimmune Genese angenommen. Der Begriff sekundäre FSGS beschreibt die histopathologische Endstrecke eines Schädigungsprozesses der Podozyten infolge zahlreicher unterschiedlicher Mechanismen, wie die Virus-assoziierte FSGS, die Medikamenten-induzierte FSGS und die (mal)adaptive FSGS. Letztere ist wohl die häufigste Form, verursacht durch eine chronische Überlastung eigentlich intakter Nephrone, entweder durch eine reduzierte absolute Anzahl (chronische Glomerulopathie, Frühgeburtlichkeit, (funktionelle) Einnierigkeit …) oder durch eine anhaltende Überbeanspruchung sonst gesunder Nieren (z. B. Diabetes mellitus und Adipositas) [1]. Bereits die klinische Manifestation ermöglicht oft eine erste Unterscheidung, da beim Fehlen eines nephrotischen Syndroms im engeren Sinn (Fehlen von Ödemen, normales oder nur leicht erniedrigtes Serum Albumin) eine sekundäre Ursache einer FSGS wahrscheinlicher ist.

Ob die primäre FSGS als solche ein eigenständiges Krankheitsbild darstellt oder besser eine pathogenetisch-definierte Bezeichnung als „primäre Podozytopathie“ oder „Autoimmunpodozytopathie“ gemeinsam mit der Minimal-Change Glomerulopathie (MCD) geprägt werden sollte, wird fortwährend diskutiert und ein Paradigmenwechsel zeichnet sich ab (Verweis auf die ÖGN-Leitlinie: MCD) [2]. Derzeit folgen maßgebliche Guidelines allerdings weiterhin der historischen Einteilung.

Die Häufigkeit der FSGS variiert geographisch stark und findet sich in Europa in etwa 9 % aller durchgeführten Nierenbiopsien bei Erwachsenen [3]. Die primäre FSGS ist die zweithäufigste Ursache eines nephrotischen Syndroms im Erwachsenenalter.

Supportive Maßnahmen zur Reduktion des intraglomerulären Drucks und der Proteinurie haben einen erwiesenen prognostischen Benefit zum Erhalt der Nierenfunktion bei allen Formen der FSGS. Demgegenüber kommen immunsuppressive Maßnahmen wie Glukokortikoide (GC) und Calcineurin-Inhibitoren (CNI) bei der primären FSGS zur initialen Therapie zum Einsatz und sollten bei sekundären und genetischen Formen vermieden werden. Komplizierte Verläufe umfassen steroid-abhängige (SA), steroid-resistente (SR) und häufig relapsierende (HR) Formen. Diese erfordern den Einsatz alternativer, steroidsparender Immunsuppressiva. Insbesondere bei SR-FSGS, frühem Erkrankungsalter und familiärer Belastung müssen genetische Ursachen in der Abklärung berücksichtigt werden. Das Risiko einer progressiven Nierenfunktionsverschlechterung und terminaler Niereninsuffizienz ist abhängig von der zugrundeliegenden Ursache und dem Therapieansprechen. Eine Rekurrenz nach Nierentransplantation betrifft vor allem die primäre FSGS und ist therapeutisch oftmals komplex.

## Pathogenese

Die Schädigung des Podozyten (Podozytopathie) ist bei der Krankheitsentstehung aller FSGS-Formen das zentrale und gemeinsame Element. Podozyten sind post-mitotische, terminal differenzierte Epithelzellen und bilden durch ihre vielzähligen Fußfortsätze, welche maschenartig ineinandergreifen und durch die sogenannte Schlitzmembran miteinander verbunden sind, einen wesentlichen Bestandteil des glomerulären Filters. Eine Podozytenschädigung führt als morphologisch frühestes Korrelat zur Abflachung der Fußfortsätze mit Verlust der Schlitzmembranen, was in der Regel mit einer schweren Proteinurie einhergeht.

Die Podozytenschädigung kann durch einen alleinigen Auslöser, beispielsweise bei monogenetischen Formen (siehe Abschnitt *Genetische Abklärung*), oder durch das Zusammenspiel mehrerer Risikofaktoren, entstehen.

Bei der primären FSGS wird als Initialereignis von einer immun-mediierten, direkten Podozytenschädigung ausgegangen. Hierfür spricht vor allem das Ansprechen auf immunsuppressive Maßnahmen, wobei auch direkte podozytenstabilisierende Effekte von GC, CNI und Rituximab beschrieben wurden und der Beweis einer zugrundeliegenden Autoimmunität bis zuletzt ausständig ist [2, 4, 5]. Einige Beobachtungen, wie beispielsweise eine unmittelbare und oft fulminante Rekurrenz der Grunderkrankung direkt nach Nierentransplantation sowie das therapeutische Ansprechen auf Plasmaaustausch, sprechen für das Vorliegen eines zirkulierenden Permeabilitätsfaktors, beispielsweise eines Zytokins [6]. Über die Jahre wurden verschiedene potenzielle Permeabilitätsfaktoren postuliert und untersucht, jedoch konnte bis dato kein auslösendes Molekül bestätigt werden [7]. Der jüngste Nachweis von krankheitsauslösenden anti-Nephrin Autoantikörpern bei PatientInnen mit MCD (Verweis auf die ÖGN-Leitlinie: MCD) hat auch Relevanz für unser Verständnis der FSGS. So konnte dieselbe Arbeitsgruppe am Fallbeispiel einer FSGS-Patientin ursächliche anti-Nephrin Autoantikörper im Rahmen einer frühen FSGS-Rekurrenz nach Nierentransplantation identifizieren [8].

Eine Vielzahl sekundärer Krankheitsauslöser wird in der Literatur beschrieben und ist in Tab. 1 dargestellt.

**Table 1:** 

Kategorie	Beispiele (Auswahl)	Screening
Medikamente, Toxine	Heroin, Kokain, anabolische Steroide, Interferon, Lithium, Pamidronat, Sirolimus, CNI, antivirale Therapie (Ledipasvir, Sofosbuvir)	Anamnese, Toxinnachweis
Infektionen	*HIV*, CMV, SARS-CoV‑2 (bei gleichzeitig vorliegender APOL1 Risikokonstellation), Parvovirus B19, EBV, HCV, Schistosoma mansoni, Filaria (Loa loa)	Anamnese + Virus-Serologie/-PCR
Maladaptiv	Reflux-Nephropathie, Nephrektomie, Renale Dysplasie, unilaterale renale Agenesie, Oligomeganephronie, niedriges Geburtsgewicht, Sichelzell-Krankheit *Fortgeschrittene glomeruläre Erkrankung*, Adipositas, maligne Hypertonie, Sichelzell-Krankheit, Schlafapnoe, zyanotisch-kongenitale Herzerkrankung, Nierenarterienstenose, Cholesterinembolie-Syndrom, Alport Syndrom	Anamnese, Status, Bildgebung

*CNI* Calcineurin Inhibitor, *HIV* humanes Immundefizienzvirus, *CMV* Zytomegalievirus, *SARS-CoV‑2* schweres-akutes-Atemwegssyndrom-Coronavirus Typ 2, *EBV* Epstein-Barr-Virus, *HCV* Hepatitis-C-Virus, *PCR* Polymerase-Kettenreaktion

Im Verlauf der Erkrankung kommt es zur kompensatorischen Hypertrophie der Podozyten und aufgrund der zunehmenden Scherkräfte konsekutiv zur Ablösung von der Basalmembran (Podozytenverlust). Ob eine Podozytenschädigung zum Krankheitsbild einer FSGS führt, hängt von mehreren Faktoren ab. Kann die auslösende Ursache gestoppt werden und funktionieren die Reparaturmechanismen, kann es zur Ausheilung kommen. Parietale Epithelzellen der Bowman-Kapsel stellen einen begrenzten Pool podozytärer Vorläuferzellen dar, aus welchem der Podozytenverlust teilweise ersetzt werden kann. Dieses Prinzip ist gültig für alle primären Podozytopathien. Bleibt die schädigende Ursache jedoch bestehen und/oder persistieren weitere maladaptive Prozesse, die eine Regeneration verhindern, kommt es zur glomerulären Vernarbung und FSGS. Mögliche maladaptive Prozesse sind beispielsweise eine unkontrollierte schwere Proteinurie oder ein anhaltend hoher intraglomerulärer Druck (mechanischer Stress), die eine adäquate Regeneration aus den Vorläuferzellen verhindern [2].

## Diagnostik und Pathologie

Die Basisdiagnostik zur Abklärung einer Proteinurie im nephrotischen Bereich erfolgt analog zur MCD (Verweis auf die ÖGN-Leitlinie: MCD). Explizit sei noch auf die seltene, insbesondere im höheren Lebensalter aber relevante Assoziation eines histologischen FSGS-Musters mit monoklonalen Gammopathien hingewiesen, daher sollten neben einer Elektrophorese auch eine Immunfixation erfolgen sowie freie Leichtketten bestimmt werden [13].

Die differentialdiagnostische Abklärung einer FSGS kann ausschließlich durch histologisch-klinische Korrelation gestellt werden. Es ist essentiell, segmentale Vernarbungen, z. B. nach Glomerulonephritis oder thrombotischer Mikroangiopathie, die nicht auf einer podozytären Störung beruhen, diagnostisch abzugrenzen, was in den meisten Fällen durch das Fehlen einer signifikanten Proteinurie leicht gelingt. Als nächster Schritt ist die Differenzierung zwischen primären, sekundären und genetischen Formen absolut zentral und stellt oftmals eine große Herausforderung dar. Klinische und histologische Charakteristika, sowie das therapeutische Ansprechen helfen bereits bei der Unterscheidung (siehe Tab. 2) und werden in den folgenden Kapiteln näher betrachtet. Die primäre FSGS ist eine Ausschlussdiagnose, sodass zunächst alle potenziellen sekundären Ursachen (Medikamente, Infektionen, maladaptive Formen) geprüft werden müssen. Wann eine genetische Abklärung erfolgen sollte, wird separat in Abschnitt *Genetische Abklärung* besprochen.

**Table 2:** 

	Primär	Genetisch	Sekundär		Unbestimmt
Ursachen/Beispiele	Autoimmun? Zirkulierender Faktor?	Familiär, sporadisch, syndromal	Viral, Medikamente	Maladaptive Prozesse	Unbestimmt
Auftreten	Akut	Variabel	Je nach Ursache	Schleichend	–
Klinik	Nephrotisches Syndrom	Variabel	Variabel	Proteinurie ohne Hypalbuminämie (kein Nephrotisches Syndrom!)	Variabel
Pathologie	LM: variabel	LM: variabel	LM: variabel	LM: Glomeruläre Hypertrophie, perihiläres Muster	–
	EM: Diffuse FPE (> 80 %)	EM: Variabel (diffuse/segmentale) FPE	EM: Variabel (segmentale/diffuse) FPE	EM: segmentale FPE (< 50 %)	EM: Segmentale FPE
IS-Ansprechen	Möglich	Keines	Keines	Keines	Variabel

*LM* Lichtmikroskopie, *EM* Elektronenmikroskopie, *FPE* Podozytenfußfortsatz-Verschmelzung

Nicht selten muss die initiale Verdachtsdiagnose im Verlauf, bspw. nach Therapieansprechen re-evaluiert werden (siehe Algorithmus/Therapie).

### Pathologie

Das namensgebende lichtmikroskopische Muster ist charakterisiert durch Vernarbungen (Sklerosierungen) von Anteilen, aber nicht der gesamten glomerulären Kapillarschlinge (segmental; < 50 %), mancher, aber nicht aller Glomeruli (fokal; < 50 %). Zur Unterscheidung von der MCD, der wichtigsten Differenzialdiagnose, ist eine ausreichend große Gewebeprobe notwendig (mindestens 10 Glomeruli!), dennoch ist das Risiko einzelne sklerosierte Glomeruli zu verpassen gegeben („sampling error“) [9]. Übrige lichtmikroskopische und elektronenmikroskopische Kriterien unterscheiden sich zwischen den beiden Entitäten nicht (Verweis auf die ÖGN-Leitlinie: MCD).

Die Columbia-Klassifikation der FSGS wurde 2004 publiziert und unterscheidet anhand des lichtmikroskopischen Sklerosierungsmusters fünf Varianten (tip lesion, perihilär, zellulär, kollapsierend und „nicht weiter spezifiziert“). Diese Kriterien weisen jedoch eine unzureichende Korrelation mit dem klinischen Phänotyp und der zugrundeliegenden Pathophysiologie auf, sodass in der aktuellen KDIGO Guideline eine Änderung der Nomenklatur (siehe Tab. 2) vorgeschlagen wird [10]. Gewisse lichtmikroskopische (Sklerosierungsmuster und Ausmaß) und elektronenmikroskopische Merkmale wie die Podozyten-FPE können bei der Unterscheidung zwischen unterschiedlichen FSGS-Formen zwar hilfreich sein, haben für sich allein jedoch unzureichenden prädiktiven Wert (siehe Tab. 2). Eine nur segmentale FPE (< 50 %) ist sehr untypisch für eine primäre FSGS und sollte an sekundäre oder genetische Formen denken lassen [1, 14]. Umgekehrt schließt das Bild einer diffusen FPE (> 80 %) sekundäre und genetische Ursachen nicht vollständig aus [1, 10]. Gelegentlich zeigen sich lichtmikroskopisch indirekte Hinweise für die zugrundeliegende Ursache einer sekundären FSGS, wie z. B. der Nephronverlust durch schwere Arteriosklerose, eine andere Glomerulopathie oder möglicherweise auch eine interstitielle Nephritis. Beim Bild der „collapsing“ Variante muss an bestimmte exogene Auslöser (bspw. HIV, COVID-19, SLE, Pamidronat) gedacht werden [15–17].

## Klinik

Bei einer FSGS ist in Abhängigkeit der zugrundeliegenden Ursache ein nephrotisches Syndrom zwar häufig, aber nicht obligat. Bei der primären Form manifestiert sich die Erkrankung oft abrupt und (zwischen etwa 50 und 90 %) [1] mit dem Vollbild eines nephrotischen Syndroms, während Personen mit sekundärer FSGS oft trotz großer Proteinurie keine vergleichbar ausgeprägten Ödeme aufweisen, und das Serum-Albumin in der Regel nicht vermindert ist. Eine Mikrohämaturie ist bei allen Formen nicht ungewöhnlich. Das Risiko für venöse Thrombembolien ist auch bei der FSGS erhöht.

Neben einer sorgfältigen Medikamentenanamnese sollten etwaige Frühgeburtlichkeit sowie relevante urologische Vorerkrankungen (unilaterale Nierenagenesie, vesiko-urethraler Reflux (VUR) …) erhoben werden. Phänotypische Auffälligkeiten (Hypakusis; Augen‑, Haut- und Bindegewebspathologien; Polydaktylie; unklare, prämature Kardiomyopathien …) sollten genau charakterisiert und durch eine detaillierte Familienanamnese (Stammbaum) ergänzt werden, um gegebenenfalls eine gezielte molekulargenetische Analyse veranlassen zu können (siehe Abschnitt *Genetische Abklärung*).

## Prognose

Das Ausmaß der Proteinurie zum Zeitpunkt der Diagnosestellung und im weiteren Verlauf das Ansprechen auf eine immunsuppressive Therapie stellen die zwei bedeutendsten Prognoseparameter dar. Während beim Erreichen einer kompletten Remission nur etwa 10 % der PatientInnen nach 10 Jahren eine dialysepflichtige Nierenerkrankung erleidet, tritt diese bei Nichtansprechen in mehr als der Hälfte aller Fälle ein [18, 19]. Die Proteinurie-Reduktion kann als prognosebestimmender Surrogatparameter für das Therapieansprechen herangezogen werden und korreliert invers mit dem künftigen Nierenfunktionsverlust [20].

## Therapie

Ähnlich wie bei der MCD fehlen auch bei der primären FSGS bei Erwachsenen randomisiert kontrollierte Studien als Evidenzbasis für die Therapieplanung. Die Evidenz ist aus Beobachtungsstudien abgeleitet bzw. aus pädiatrischen Untersuchungen extrapoliert. Erschwerend kommt hinzu, dass in diesen Studien oftmals primäre, sekundäre und genetische FSGS-Formen undifferenziert als eine Kohorte eingeschlossen wurden. Bei Letzteren ist eine immunsuppressive Therapie nicht indiziert, in diesen Fällen steht die Supportivtherapie im Mittelpunkt der therapeutischen Überlegungen (siehe unten).

**Figure 1:**
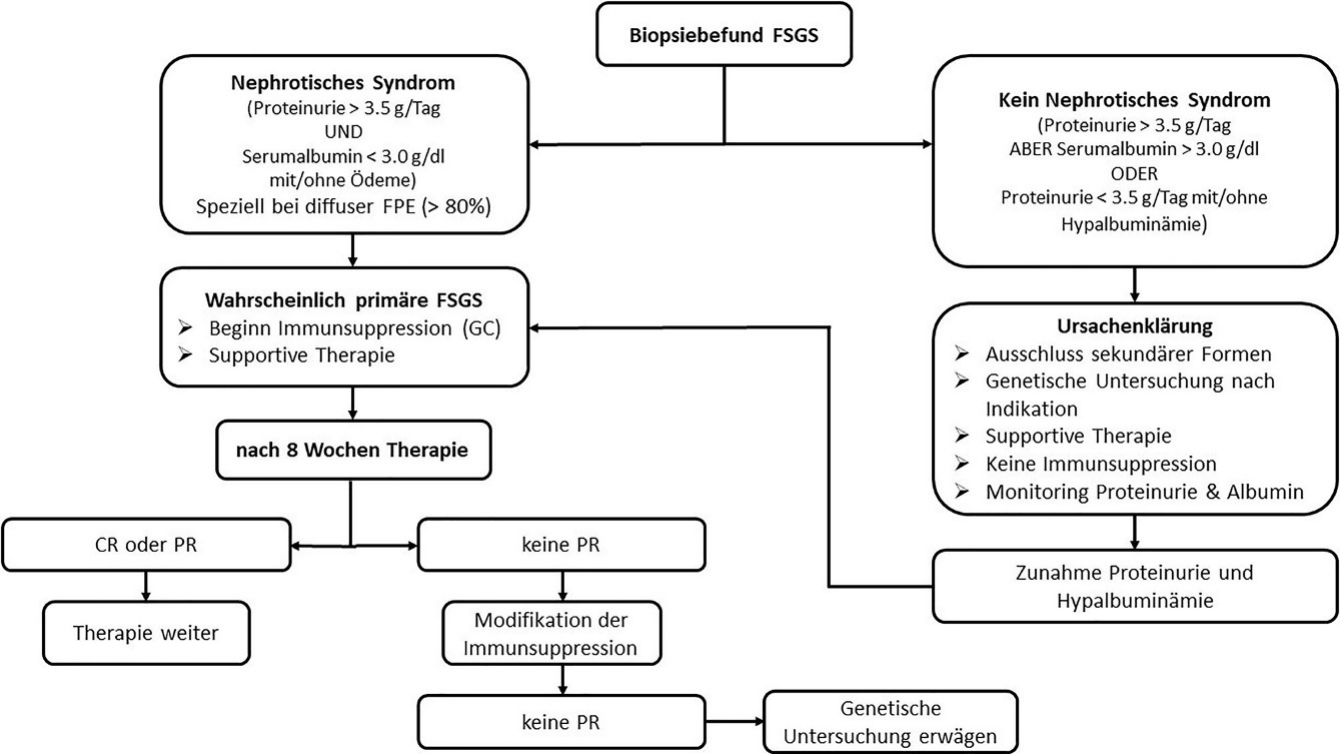

Ein möglicher Algorithmus ist in Abb. 1 dargestellt.

### Immunsuppressive Therapie der primären FSGS

Nur die primäre FSGS mit nephrotischem Syndrom qualifiziert sich für eine immunsuppressive Therapie. Bei einer geringen Spontanremissionsrate wird eine immunsuppressive Therapie aufgrund der mit einer Therapie-induzierten Remission einhergehenden renalen Prognoseverbesserung empfohlen [19].

Das therapeutische Ansprechen wird nach folgenden Kriterien beurteilt (Tab. 3).

**Table 3:** 

	Proteinurie	eGFR	Serumalbumin
*Komplette Remission (CR)*	< 0,3 g/Tag oder < 0,3 g/g Kreatinin	Stabil	Im Normbereich (> 3,5 g/dl)
*Partielle Remission (PR)*	0,3–3,5 g/Tag oder 0,3–3,5 g/g Kreatinin, jeweils mit > 50 % Albuminuriereduktion von Baseline	Stabil	–

*eGFR* geschätzte glomeruläre Filtrationsrate

In Analogie zur MCD und gemäß den KDIGO-Leitlinien empfehlen wir die Erstmanifestation einer primären FSGS mit GC zu behandeln. Am gebräuchlichsten sind Methylprednisolon und Prednisolon (4 mg Methylprednisolon entsprechen 5 mg Prednisolon).

- Umrechnung Prednisolon zu Methylprednisolon: Prednisolondosis: 5 × 4
- Umrechnung Methylprednisolon zu Prednisolon: Methylprednisolondosis: 4 × 5
- Prednisolon in einer Tagesdosis von 1 mg/kg/d (Maximaldosis 80 mg/d). Die Einnahme sollte morgens zwischen 7 und 8 Uhr als Einzelgabe erfolgen. Das alternative alternierende Schema mit 2 mg/kg/d (Maximaldosis 120 mg/d jeden 2. Tag) ist in Österreich nicht gebräuchlich. Aufgrund der ödem-bedingten passageren Gewichtszunahme sollte dabei das Körpergewicht vor Krankheitsmanifestation als Ausgangswert herangezogen werden.
- Die Mindestdauer dieser GC-Hochdosistherapie beträgt vier Wochen bis zum Erreichen einer CR (nach KDIGO), sollte aber maximal 16 Wochen fortgeführt werden.
- In Analogie zur MCD sehen wir jedoch eine derartig lange Hochdosis-GC Therapie bis zu 16 Wochen, die mit erheblicher Toxizität vergesellschaftet ist, kritisch. Bei ausbleibender Remission oder Auftreten von therapieassoziierten Nebenwirkungen sollten schon frühzeitig alternative Behandlungsstrategien (siehe unten) in Erwägung gezogen werden.
- Nach Erreichen einer CR soll die GC-Therapie in unveränderter Dosierung noch für zwei weitere Wochen fortgeführt und danach – bei anhaltendem Response – schrittweise dosisreduziert werden („Tapering“).
- Bei Ausbleiben einer CR aber Erreichen einer PR innerhalb von acht Wochen, empfehlen wir das Fortsetzen der Steroidtherapie in modifizierter Dosis (siehe rechter Tapering-Schenkel; MCD-Kapitel, Abb. 1).
- Als mögliches Dosisreduktionsschema empfehlen wir die im MCD-Kapitel (Abb. 1) dargestellte Vorgehensweise.
- Ist nach acht Wochen Hochdosis-GC Therapie kein Ansprechen erkennbar, ist ein Behandlungserfolg durch eine fortgesetzte GC-Monotherapie im Hochdosisbereich unwahrscheinlich, sodass wir hier frühzeitig alternative Behandlungsmethoden, insbesondere den Einsatz von CNI befürworten.
- Als Gesamtdauer der GC-Behandlung werden 24 Wochen empfohlen.

Definitionsgemäß wird erst beim Ausbleiben eines Ansprechens (CR oder PR) nach 16 Wochen Therapiedauer mit Hochdosis GC-Therapie von einer steroid-resistenten primären FSGS (SR-FSGS) gesprochen. Eine derartige lange Hochdosis-Steroidexposition sollte unseres Erachtens jedoch vermieden werden. Kommt es zum Rezidiv während des GC-Taperings, oder innerhalb von zwei Wochen nach Beendigung der GC-Therapie, spricht man von steroidabhängiger FSGS (SA FSGS). Ein Rezidiv (oder Relaps) definiert sich durch eine Proteinurie > 3,5 g/Tag oder > 3,5 g/g Kreatinin nach zuvor erreichter Remission. Bei zwei oder mehr Rezidiven innerhalb von sechs Monaten oder > vier Rezidiven pro Jahr wird von häufig relapsierender (HR) FSGS gesprochen.

Für PatientInnen mit relativer oder absoluter Kontraindikation gegenüber einer GC-Therapie oder GC-Unverträglichkeit wird eine CNI-Therapie als Initialtherapie empfohlen. Dabei können beide CNI, Tacrolimus (TAC) und Cyclosporin A (CsA), eingesetzt werden. Rituximab (RTX) stellt eine mögliche second-line Alternativtherapie bei Kontraindikationen/Unverträglichkeit oder limitierender Nebenwirkung der CNI dar. Diese Therapieoptionen werden auch für die SA-FSGS als Steroidersatz sowie bei der SR-FSGS empfohlen.

Die Auswahl erfolgt nach lokaler Erfahrung, Begleiterkrankungen sowie PatientInnenwunsch.

Mögliche Schemata sind:

- TAC 0,05–0,1 mg/kg/d als Einmaldosis in retardierter Formulierung oder unretardiert in zwei geteilten Tagesdosen in 12-Stundenabstand; möglicher Talspiegel: 5–10 ng/ml [10, 21]. Begleitend kann auch eine GC-Therapie in niedrigerer Dosis (0,5 mg/kg) mit Tapering ab Woche 9 gegeben werden [22].
- CsA 3–5 mg/kg/d in zwei geteilten Tagesdosen in 12-Stundenabstand; Talspiegel: 100–175 ng/ml. Begleitend kann auch eine Niedrigdosis-GC-Therapie in Anlehnung an die Originalarbeit zur CsA-Therapie bei SR-FSGS von Cattran et al. [23] mit 0,15 mg/kg (maximal 15 mg/d) für sechs Monate gegeben werden.

Beide CNI sollten zumindest für 4–6 Monate unter Erreichen adäquater Talspiegel angewendet werden, um die Effektivität dieser Therapie zu beurteilen, und bevor im Falle eines fehlenden Ansprechens von einer CNI-Resistenz gesprochen wird. Die CNI-Therapie sollte im Falle eines klinischen Ansprechens (CR oder PR) für mindestens ein Jahr in der zur Remission führenden Dosis fortgeführt und nachfolgend langsam und schrittweise über 6 bis 12 Monate dosisreduziert werden. Auch Änderungen in der glomerulären Filtrationsrate spielen für die Dosisfindung eine Rolle; bei steigendem Kreatinin (> 30 % von Baseline) wird eine Dosisreduktion des CNI empfohlen, bei einer eGFR < 30 ml/min/1,73 m^2^ ist ein Absetzen der CNI-Therapie zu diskutieren [10]. Trotz guter Effektivität in der Remissionsinduktion bei PatientInnen mit SR-FSGS kann es häufig (bei 40–70 % innerhalb eines Jahres) zu einem Rezidiv nach dem Absetzen kommen [23, 24].

TAC zeichnet sich durch ein im Vergleich zu CsA günstigeres Nebenwirkungsprofil aus und wird deshalb heute allgemein bevorzugt. Beide Substanzen zeigen jedoch z. T. unterschiedliche Nebenwirkungen, sodass individualisiert ein Wechsel angezeigt sein kann.

Als Zweitlinientherapie stehen folgende Optionen zur Verfügung:

- Rituximab kann als Therapieoption bei CNI-Abhängigkeit mit ungünstigem Nebenwirkungsprofil, CNI-Resistenz oder FR-FSGS nach CNI-Absetzen gewählt werden [25]. Ein einheitliches Dosisregime ist nicht etabliert.
  Wird eine anti-CD20-Therapie noch im manifesten nephrotischen Syndrom verabreicht, kann der renale Verlust des Wirkstoffs eine frühere neuerliche Gabe erforderlich machen. Aus diesen Gründen halten wir die RTX-Dosierung aus dem TURING-Protokoll (2 × 1 g im Abstand von zwei Wochen) für zweckmäßig (Details zu einer Therapie mit RTX: Separater Beitrag „Allgemeine Empfehlungen für die Behandlung glomerulärer Erkrankungen“). Für die weitere Dosierung kann in Anlehnung an andere Indikationen entweder eine intervallfixierte (z. B. alle sechs Monate) oder klinisch gesteuerte (Anstieg der Proteinurie ± CD19^+^ Zellzahl) Dosierung (jeweils Einzelgaben à 500 oder 1000 mg) für zwei Jahre in Betracht gezogen werden.

**Figure 2:**
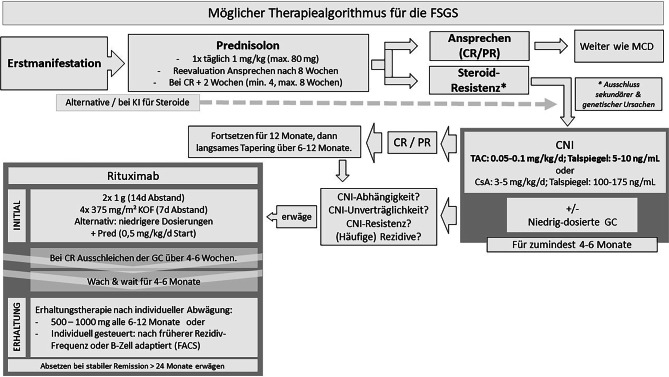

Die beschriebenen Therapieoptionen sind zur besseren Übersicht als Therapiealgorithmus in Abb. 2 zusammengefasst.

### Supportivtherapie

Die unten angeführten Komponenten der Supportivtherapie sollten bei allen PatientInnen mit FSGS optimiert werden, bei sekundärer FSGS stehen sie neben einer möglichen Kausaltherapie des ursächlichen Auslösers im Mittelpunkt. Diese Supportivmaßnahmen umfassen:

1. Hemmer des Renin-Angiotensin-Systems (RASi) in maximal tolerierter Dosierung.
2. Antihypertensive Therapie mit systolischem Zielblutdruck < 120 mm Hg, sofern verträglich.
3. Absolute Nikotinkarenz
4. Anstreben von Normalgewicht
5. Kochsalzbeschränkung
6. Aus pathophysiologischen Überlegungen und basierend auf ersten Daten der DAPA-CKD-Subanalyse zeichnet sich auch für die sekundäre FSGS eine Indikation für die SGLT2-Hemmer zusätzlich zur Basistherapie mit einem RASi ab [26]. Weitere Daten sind aus der EMPA-KIDNEY Studie zu erwarten.

## Genetische Abklärung

Seit den 1990er-Jahren erlangen hereditäre Ursachen eines FSGS-Läsionsmusters zunehmende Aufmerksamkeit. Mittlerweile sind Mutationen in über 60 Genen bekannt, die mit diesem histologischen Bild vergesellschaftet sind, die Liste wächst laufend [1, 27].

Die Häufigkeit einer erblichen Ursache einer FSGS variiert in Abhängigkeit vom Manifestationsalter stark [2]. Während einem kongenitalen nephrotischen Syndrom in den meisten Fällen eine hereditäre Genese zugrunde liegt, findet man im Jugend- und Erwachsenenalter nur noch in 10–20 % der Fälle pathogene Varianten. Bei bestimmten klinischen Merkmalen ist die Wahrscheinlichkeit erhöht, eine FSGS nachzuweisen [28]. Die entsprechenden Gene kodieren größtenteils für Proteine, welche für eine Vielzahl unterschiedlicher Podozytenfunktionen relevant sind, darunter Bestandteile der Schlitzmembran, Kernproteine, Transkriptionsfaktoren und tRNA-modifizierende Proteine, Zytoskelettproteine, Bestandteile der glomerulären Basalmembran, Proteine der mitochondrialen Coenzym Q10-Biosynthese sowie lysosomale und andere metabolische Proteine [2]. Die Vererbung ist in den meisten Fällen autosomal-rezessiv, mitunter aber auch autosomal-dominant oder X‑chromosomal.

Der Großteil der kongenitalen Formen eines nephrotischen Syndroms wird durch Mutationen in einer Handvoll Gene verursacht, am häufigsten *NPHS1* (Nephrin), *NPHS2* (Podocin) und *WT1* (Wilms tumor 1). Im Erwachsenenalter stehen Mutationen im Typ 4-Kollagen-Gen *COL4A* (auch Ursache des Alport-Syndroms) im Vordergrund. Bei PatientInnen afrikanischer Herkunft sollten zusätzlich immer zwei Risikoallele im *APOL1*-Gen (kodiert für Apolipoprotein L1) getestet werden, die die Wahrscheinlichkeit für das Auftreten einer FSGS in Kombination mit anderen Risikofaktoren (z. B. Hypertonie) deutlich erhöhen. Insgesamt gibt es aber beträchtliche Überlappungen über die Altersgruppen. Genetische FSGS-Formen, die im Kindesalter auftreten, werden häufig autosomal-rezessiv vererbt, die genetische FSGS des Erwachsenenalters nicht selten autosomal-dominant. Phänomene wie inkomplette Penetranz und modifizierende Gene erschweren die Interpretation der Bedeutung genetischer Varianten in einer Familie zusätzlich. Zu berücksichtigen ist speziell auch die Art der Mutation (z. B. Funktionsverlust vs. dominant negativ), welche erheblichen Einfluss auf den renalen Phänotyp, den Zeitpunkt des Auftretens der Erkrankung oder den Erbgang haben kann. Nicht zuletzt sind syndromale Formen der Nephropathie in Kombination mit anderen Organmanifestationen von nicht-syndromalen Formen zu unterscheiden. Es kann davon ausgegangen werden, dass die Prävalenz primär genetischer FSGS-Formen im Erwachsenenalter unterschätzt wird, zumal die klinischen und histologischen Phänotypen in der Regel weder von einer primären noch von einer sekundären FSGS zu unterscheiden sind. Auch das Ausmaß der Fußfortsatzabflachung ist bei den genetischen Formen durchaus variabel und kann diffus oder nur segmental sein, weshalb die Histologie nur bedingt zur Abgrenzung von immun-mediierten Formen der FSGS hilft [1, 28].

Neben dem kongenitalen nephrotischen Syndrom ist eine genetische Diagnostik bei entsprechender Familienanamnese sowie bei FSGS mit syndromalen Charakteristika (s. oben) etabliert. Auch im Kontext einer Lebendnierenspende bei PatientInnen mit FSGS ist ihr Stellenwert mittlerweile anerkannt. Allerdings schließt eine unauffällige Familienanamnese eine erbliche Ursache einer FSGS keineswegs aus (s. oben). Parallel zu den methodischen Fortschritten in der Molekulargenetik und den sinkenden Kosten zeichnet sich daher ein liberalerer Zugang zur genetischen Testung ab, insbesondere bei steroidresistenten Verläufen bzw. ausbleibendem Ansprechen auf eine andere immunsuppressive Therapie [29]. Bei Zunahme der Genanalysen wird sich zwangsläufig eine Häufung unklarer Befunde (Varianten unklarer Signifikanz, VUS) ergeben. Im Einzelfall kann daher eine Zuordnung, ob es sich tatsächlich um eine klinisch relevante Variante handelt, schwierig sein.

Für die genetische Diagnostik werden inzwischen massiv-parallele Sequenzierverfahren verwendet, bei der meist alle proteinkodierenden Gene des Erbguts (Exom) potenziell erfasst werden. Die Auswertung erfolgt typischerweise über virtuelle diagnostische Panels, bei der nur die potenziell relevanten Gene betrachtet werden. Je nach Analysestrategie, gewünschtem Aufwand und Vollständigkeit unterscheidet sich die Anzahl der untersuchten Gene. Verfügbare Panels beinhalten durchwegs die Gene *NPHS1, NPHS2, WT1, PLCE1 *(die am häufigsten betroffenen Gene beim kongenitalen nephrotischen Syndrom) sowie die „Alport-Gene“ *COL4A3, COL4A4 *(beide autosomal) sowie *COL4A5* (X-chromosomal). War früher ein stufenweises Vorgehen die Regel (beispielsweise zuerst eine Untersuchung einiger weniger Gene wie *NPHS2, ACTN4, TRPC6 *und *INF2*), ermöglichen moderne Verfahren eine gleichzeitige und daher rasche Analyse gut charakterisierter „FSGS-Gene“ [27, 30]. Bei dringendem Verdacht auf eine genetische Ursache bei gut charakterisierten PatientInnen kann die Analytik auch beliebig auf viele oder „alle“ Gene ausgedehnt werden, wobei die Interpretation der dann unweigerlich gefundenen Varianten unklarer Signifikanz eine gute interdisziplinäre Zusammenarbeit und Fallbesprechungen voraussetzt.

Eine genetische Diagnose hat wichtige praktische Konsequenzen. Sie beendet diagnostische Odysseen, erlaubt das Absetzen einer immunsuppressiven Therapie bzw. verhindert, dass eine solche überhaupt begonnen wird, kann eine bessere Prognoseeinschätzung ermöglichen und führt zur gezielten Suche nach assoziierten extrarenalen Manifestationen. Auch beim Angehörigenscreening ist eine molekulargenetische Untersuchung wichtig, vor allem im Kontext einer Lebendspende. Schließlich existiert in seltenen Fällen eine spezifische Therapie (Glomerulopathien mit Störungen der Coenzym-Q10 Biosynthese, vor allem bei Kindern und Jugendlichen) [31].

Indikationen für eine molekulargenetische Abklärung (modifiziert nach [27, 28])

- FSGS im Erwachsenenalter mit positiver Familienanamnese
- V. a. primäre FSGS resistent auf Immunsuppression
- Sekundäre FSGS ohne offensichtliche Ursache

## Transplantation

Die primäre FSGS weist nach einer Nierentransplantation eines der höchsten Rezidivrisiken aller Glomerulopathien auf. Etwa ein Drittel der PatientInnen erleidet eine Rekurrenz im Transplantat [32].

Ein Rezidiv kann früh (binnen Stunden oder weniger Tage) auftreten, aber auch erst nach Monaten und mitunter Jahren, wobei mit zunehmendem zeitlichem Abstand zur Transplantation die Unterscheidung primäre FSGS versus sekundäre FSGS schwieriger wird. Histologisch zeigt sich bei frühen Formen eine Podozytopathie mit typischerweise diffuser Fußfortsatzabflachung bei oft (noch) normalem lichtmikroskopischen Befund.

Der wichtigste Risikofaktor für eine Post-Transplant-Rekurrenz ist eine bereits stattgehabte Rekurrenz in einem vorherigen Nierentransplantat. In diesem Fall beträgt das Risiko einer neuerlichen Rekurrenz 80 % bei der folgenden Nierentransplantation [33]. Als weitere prädisponierende Faktoren sind junges Alter bei Erstmanifestation und rasch-progredienter Verlauf anerkannt. Umgekehrt erlauben diese klinischen Faktoren im Einzelfall keine ausreichende Risikoeinschätzung und haben daher auch keine therapeutische Konsequenz im Sinne einer präemptiven Therapie [34].

Eine FSGS-Rekurrenz geht, verglichen mit transplantierten FSGS-PatientInnen ohne Rekurrenz, mit einem etwa fünffach erhöhten Risiko für einen Transplantatverlust einer [32]. Die Prognose wird im Wesentlichen durch das Therapieansprechen determiniert, für welches es wiederum keine eindeutigen Empfehlungen gibt. Die Behandlungsoptionen umfassen den Einsatz von RTX sowie Apherese-Verfahren, die Datenlage zu neueren B‑Zell-depletierenden Substanzen ist limitiert und uneinheitlich [35, 36]. In Einzelfällen benötigen PatientInnen chronisch extrakorporale Therapieverfahren [32]. Grundsätzlich sollten diese PatientInnen an einem Zentrum mit entsprechender Expertise behandelt werden.
